# Negative workplace gossip and turnover intention among Chinese kindergarten teachers: the mediating role of work engagement

**DOI:** 10.3389/fpsyg.2026.1824988

**Published:** 2026-06-24

**Authors:** Lili Xu, Baoan Feng, Mei Luo

**Affiliations:** 1 College of Teacher Education, Quzhou University, Zhejiang, China; 2 College of Performing Arts and Education, Zhejiang Vocational Academy of Art, Hangzhou, Zhejiang, China

**Keywords:** early childhood education, kindergarten teachers, negative workplace gossip, turnover intention, work engagement

## Abstract

**Introduction:**

High rates of staff attrition in early childhood education (ECE) have emerged as a worldwide concern, undermining the operational stability of educational settings and the developmental well-being of young children. Negative workplace gossip, a prevalent form of dysfunctional interpersonal conduct, represents a potential key determinant of kindergarten teachers’ intent to leave their positions, yet the underlying psychological mechanisms through which it operates remain underexplored. This study draws on Conservation of Resources (COR) theory to fill this research gap.

**Methods:**

This cross-sectional study aimed to examine the association between perceived negative workplace gossip and kindergarten teachers’ turnover intention, as well as the mediating role of work engagement. A total of 1,306 practicing kindergarten teachers in mainland China completed validated self-report questionnaires, including the Perceived Negative Workplace Gossip Scale, the Intent to Leave Scale, and the Work Engagement Scale. IBM SPSS Statistics 29.0 and Amos 31.0 were used for statistical analysis. The analytical procedures included confirmatory factor analysis (CFA) for measurement model validation, Pearson correlation analysis for bivariate relationship testing, and the PROCESS macro to verify the mediating effect.

**Results:**

Perceived negative workplace gossip showed a positive correlation with turnover intention and a negative correlation with work engagement, while work engagement was negatively correlated with turnover intention. Regression analysis further confirmed that negative workplace gossip significantly and positively predicted teachers’ turnover intention, and work engagement partially mediated the link between negative workplace gossip and turnover intention.

**Discussion:**

These findings clarify the mediating psychological pathway through which negative workplace gossip shapes kindergarten teachers’ turnover intention, advancing the theoretical framework for research on ECE teacher attrition. Practically, the results offer feasible strategies for ECE institutions to construct positive workplace environments, improve teachers’ work engagement, and lower staff turnover rates.

## Introduction

Employee turnover, a core concept in organizational behavior research, refers to the active or passive termination of the employment relationship between individual employees and their affiliated organizations or teams (Akasheh et al., 2024). Within the educational landscape, particularly in early childhood education (ECE), the persistent issue of teacher attrition has evolved into a globally recognized challenge that demands urgent attention. This phenomenon extends beyond the individual career decisions of educators, exerting profound and far-reaching consequences for the operational stability of educational institutions, the caliber of educational services delivered, and the developmental outcomes of young children (Mims et al., 2008; Wartenberg et al., 2023). From a dialectical perspective, individual turnover may bring temporary career adjustments for teachers, such as pursuing better working conditions or professional development opportunities (Fee, 2024). However, for the ECE sector characterized by labor intensity and strong continuity of care, the negative impacts of high turnover far outweigh these individual-level potential benefits.

Kindergarten teacher turnover has become a common concern across countries, with substantial variation in prevalence and patterns worldwide. In the United States, longitudinal data from publicly funded ECE programs in Louisiana show that less than 40% of teachers remained at their original institutions after 3 years, with childcare teachers, toddler teachers, and new teachers having particularly high turnover rates (Bellows et al., 2022). From 2010 to 2022, the average annual turnover rate of childcare workers in the U. S. reached 14.9%, which was 65% higher than that of typical occupations in 2022, while preschool and kindergarten teachers had a turnover rate of 8.5% (Fee, 2024). Within the Netherlands, the attrition rate among newly appointed kindergarten educators stands at approximately 15%, a figure marginally lower than that documented in English-speaking nations like the United Kingdom and Australia. However, the core drivers of this turnover, such as excessive workload and insufficient institutional support, remain aligned with global patterns (Brok et al., 2017). In Japan, a mere 30.7% of kindergarten teachers in Nagasaki Prefecture express a willingness to remain in the profession for more than 5 years, a statistic that signals a profound deficit in long-term career commitment among early childhood educators (Tayama et al., 2019). These cross-national data collectively show that kindergarten teacher turnover is a widespread problem that plagues the global ECE sector, and its severity varies slightly across regions due to differences in educational systems and working conditions.

In China, kindergarten teacher turnover is similarly severe, and it exhibits distinctive features amid the swift expansion of the preschool education sector. Analysis from a nationwide sample of 1,118 early childhood educators indicated that 43.65% had left a teaching position on at least one occasion: 25.60% reported one resignation, 10.64% had resigned twice, and 8.41% had departed from their roles three times (Ren et al., 2024). As of 2020, the attrition rate for educators employed at private kindergartens in China surged to 41% (Yang et al., 2021). Regionally divergent patterns are also evident: between 2013 and 2018, the turnover rate for kindergarten teachers in Shenzhen ranged from 12 to 14%, whereas the rate in Hubei Province hit 29.7% (Ren et al., 2024). Unlike some Western countries, Chinese kindergarten teacher turnover is significantly correlated with demographic factors such as gender and marital status—male teachers and unmarried teachers have higher turnover tendencies—and is consistently affected by occupational stress, reward levels, management mechanisms, and career aspirations (Ren et al., 2024). In addition, the imbalance between supply and demand of kindergarten teachers exacerbates the impact of turnover: although the Chinese government has promoted the popularization of preschool education since 2010, the teacher-child ratio in 2021 was 1:17, far exceeding the recommended standard of 1:9, leading to excessive workload and pressure on existing teachers and further stimulating turnover intentions (Ren et al., 2024). Overall, elevated rates of staff attrition have emerged as a substantial obstacle to the steady and sustainable advancement of early childhood education in China (Di et al., 2023).

The high turnover of kindergarten teachers has triggered a series of negative chain reactions, bringing serious harms to multiple stakeholders including children, educational institutions, and the entire ECE system. For young children in the critical period of growth and development, frequent teacher changes disrupt the establishment of stable teacher-child attachment relationships, which is not conducive to the development of their social emotions, cognitive abilities, and sense of security (Choi et al., 2019). Studies have shown that the instability of care and the lack of continuous high-quality teacher-child interactions can significantly reduce children’s school readiness, especially for low-income and ethnically diverse groups (Tran and Winsler, 2011). For kindergarten institutions, high turnover increases recruitment and training costs, disrupts the continuity of educational programs, and leads to the loss of institutional knowledge and experience, thereby affecting the overall quality of education and social reputation (Doromal et al., 2022). From a macro perspective, the continuous loss of kindergarten teachers aggravates the shortage of ECE resources, widens the gap in educational quality between urban and rural areas and between public and private institutions, and hinders the sustainable development of preschool education and the realization of educational equity goals (Di et al., 2023).

Given the profound seriousness and far-reaching consequences of kindergarten teacher turnover, pinpointing the determinants that drive this phenomenon has become a central priority for both academic research and practical stakeholders. Existing studies have explored relevant factors from multiple levels such as individual characteristics (e.g., self-efficacy and career aspirations), organizational conditions (e.g., workplace environments and institutional backing), and societal influences(e.g., social recognition and wage levels) (Hur et al., 2023; Grant et al., 2019; Ren et al., 2024; Shan-huai, 2022; Yang and Yang, 2023). Notably, most existing empirical conclusions are derived from universal or Western workplace contexts, while localized explorations targeting Chinese kindergarten teachers remain relatively limited and fragmented (Feng et al., 2025; Xu et al., 2026). Recent indigenous studies focusing on China’s unique ECE workplace culture have begun to focus on negative interpersonal workplace behaviors, confirming that workplace interpersonal quality profoundly shapes Chinese kindergarten teachers’ psychological experience and professional behavioral decisions (Zhou and Feng, 2026).

However, few studies have paid attention to the impact of workplace interpersonal interactions—specifically, detrimental workplace gossip—on kindergarten teachers’ intent to leave their positions (Feng et al., 2025; He and Wei, 2022). As a typical destructive interpersonal workplace behavior, negative workplace gossip is defined as the informal transmission of unsubstantiated critical information targeting colleagues or work groups (Chandra and Robinson, 2009). In the ECE context, such behavior specifically includes character assassination, deliberate dissemination of misleading information, unverified rumors, and the spreading of unfavorable personal or professional claims about coworkers (Feng et al., 2025). Different from Western ECE practitioners who have relatively independent work boundaries and diverse social coping resources, Chinese kindergarten teachers work in closed, high-density, and relationship-dependent workplace environments, where long-term intensive team collaboration and collectivist cultural norms make them extremely sensitive to interpersonal negative events. Empirical evidence focusing on Chinese preschool teachers confirms that these negative interpersonal acts trigger severe workplace problems, including intensified interpersonal conflicts, workplace victimization, persistent psychological stress, and emotional exhaustion, while significantly reducing teachers’ job satisfaction (He and Wei, 2022; Wang et al., 2022). From the perspective of conservation of resources (COR) theory, these chronic interpersonal stressors continuously deplete teachers’ limited emotional and social resources, undermine their positive work attitudes, and consequently increase their turnover intentions (Hobfoll et al., 2017; Rasool et al., 2021). This adverse mechanism is particularly salient in kindergarten settings. Kindergartens rely on long-term, high-frequency teacher collaboration and require intensive daily emotional labor; such working characteristics make preschool teachers more susceptible to the resource-depleting effects of toxic gossip culture (Xu et al., 2026).

Consistent with indigenous ECE research, Chinese kindergartens feature closed collective workplace relationships and unique socio-cultural norms that prioritize interpersonal harmony, further amplifying the negative impacts of workplace gossip on teachers’ psychological states and work behaviors (Xu et al., 2026). When exposed to persistent negative gossip, teachers experience continuous resource loss, which impairs their positive psychological functioning at work. For local kindergarten teachers, long-term exposure to negative gossip will continuously consume their psychological resources, weaken their work enthusiasm and professional identity, and eventually become a critical situational trigger for their turnover decisions, which explains why teacher attrition is prevalent in Chinese kindergartens beyond traditional occupational pressure factors. As a core positive psychological state, work engagement is characterized by vigor, dedication, and absorption (Dunlop and Scheepers, 2022). Existing organizational research has well documented that adverse workplace factors can diminish employee work engagement, which in turn promotes turnover intentions (Bakker and Demerouti, 2007). Despite these established associations, current indigenous ECE scholarship has only verified the general influence of workplace negative factors on teachers’ psychological outcomes. Empirical evidence clarifying the mediating role of work engagement in the linkage between negative workplace gossip and turnover intention among Chinese kindergarten teachers remains scarce (Feng et al., 2025; Zhou and Feng, 2026).

However, it remains underexplored whether work engagement serves as a mediating mechanism in the association between negative workplace gossip and kindergarten teachers’ turnover intentions. Filling this research gap is not only conducive to enriching the theoretical framework of kindergarten teacher turnover but also provides practical guidance for ECE institutions foster healthier workplace interpersonal climates, boost teacher work engagement, and lower staff turnover rates. Against this background, this study focuses on Chinese kindergarten teachers, constructs a theoretical model with negative workplace gossip as the independent variable, turnover intention as the dependent variable, and work engagement as the mediating variable, and empirically tests the internal mechanism of negative workplace gossip affecting kindergarten teachers’ turnover intentions. Different from previous Western-oriented universal research, this study focuses on the localized context of Chinese preschool education, aiming to supplement the indigenous theoretical mechanism of teacher turnover in the Chinese ECE industry. The results of this investigation are anticipated to offer fresh insights and evidence-based strategies for mitigating kindergarten teacher turnover and advancing the sustainable development of the ECE workforce.

## Theoretical background and hypotheses

Drawing on Conservation of Resources (COR) theory (Hobfoll, 1989), this research constructs a theoretical framework to unpack how Negative workplace gossip shapes turnover intention among Chinese kindergarten educators, with work engagement functioning as a mediating mechanism. COR theory posits that individuals are motivated to acquire, preserve, and safeguard valuable resources, and that psychological strain emerges when they encounter resource depletion or the prospect of resource loss (Hobfoll et al., 2017). Negative workplace gossip, as a typical adverse interpersonal burden, directly destroys individual psychological resources such as emotional energy, social trust, and psychological security in the workplace (Feng et al., 2025; Zou et al., 2020), which are especially vital for kindergarten teachers who depend on stable emotional resources and positive collegial relationships to perform their daily educational tasks. When teachers suffer from continuous resource loss caused by negative gossip, their work engagement—a vital motivational resource reflecting vigor, dedication, and absorption in work—will be significantly weakened (He and Wei, 2022; Zeng et al., 2022). In accordance with the fundamental logic of COR theory, reduced work engagement further pushes teachers to adopt withdrawal behaviors to avoid continuous resource consumption, thereby strengthening their turnover intention (Aggarwal et al., 2020; Bakker and Demerouti, 2007; Tricahyadinata et al., 2020). Therefore, this study takes COR theory as the theoretical cornerstone and regards work engagement as a key psychological resource in the process of resource loss and transmission, aiming to explore the associative pathway linking negative workplace gossip with turnover intention among kindergarten teachers.

## Negative workplace gossip and turnover intention

Negative workplace gossip has been widely identified as a detrimental interpersonal phenomenon that triggers a cascade of adverse consequences for targeted employees and organizational functioning. Prior empirical research consistently demonstrates that exposure to negative workplace gossip is associated with increased emotional distress, psychological discomfort, and diminished psychological safety, as individuals face unsubstantiated criticism, reputation damage, and social exclusion (Ullah et al., 2021; Qi et al., 2022). At the cognitive and behavioral levels, negative workplace gossip erodes employees’ sense of organizational belonging, undermines trust in colleagues and supervisors, and depletes psychological resources critical for sustaining work engagement (Parray et al., 2023; Durmuş et al., 2024). For instance, research conducted in healthcare and educational contexts have shown that employees targeted by negative gossip often experience heightened job stress, reduced occupational satisfaction, as well as a feeling of powerlessness in navigating the workplace environment (Lee et al., 2024; Mujtaba and Senathip, 2020). These negative experiences collectively drive employees to seek escape from the aversive work context, ultimately manifesting in turnover intention. From the perspective of Affective Events Theory, negative workplace gossip acts as a chronic aversive interpersonal incident that elicits sustained negative emotions (e.g., anxiety, frustration) and disrupts employees’ psychological well-being, prompting them to contemplate departure as a means to reduce psychological strain (Ullah et al., 2021). Resource Conservation Theory further explains this mechanism: negative workplace gossip depletes valuable relational and personal resources (e.g., workplace backing, self-esteem), and employees respond by intending to leave the organization to avoid further resource loss (Qi et al., 2022). Empirically, this relationship has been validated across diverse contexts—from kindergarten teachers to hospital nurses, in which greater exposure to workplace negativity is reliably associated with more elevated exit tendencies (Durmuş et al., 2024; Parray et al., 2023). Building on these theoretical frameworks and empirical findings, the following hypothesis is proposed:

*H1: Negative workplace gossip is positively related to turnover intention.*

## Work engagement as a mediating factor

Work engagement can be conceptualized as a constructive, work-centered psychological condition that is marked by three fundamental components: vitality, devotion, and full immersion in one’s work (Bakker, 2014; Schaufeli et al., 2002). Vigor is manifested in the consistent physical and mental stamina that employees maintain during work, which empowers them to persevere when confronted with workplace challenges and pressures. Dedication is demonstrated through a profound sense of engagement, self-esteem, and purpose derived from one’s professional tasks, paired with eagerness and commitment to channeling effort into work. Absorption describes a condition of complete engrossment in work activities, typified by heightened focus, challenges in disengaging from work, and a distorted perception of the passage of time (Bakker, 2014). These dimensions collectively reflect an employee’s proactive and positive orientation toward work. Work engagement exerts dual effects on individuals and organizations. Work engagement exerts dual effects on individuals and organizations. In terms of positive effects, elevated levels of work engagement are strongly linked to improvements in job performance, proactive workplace behaviors, and innovative contributions (Mazzetti et al., 2021; Puspitasari and Darwin, 2021). For kindergarten teachers, whose work involves high emotional labor and interactive demands, high work engagement fosters patience, care, and effective interaction with young children, thereby improving educational quality (Rusu and Colomeischi, 2020). Additionally, engaged employees exhibit higher job satisfaction, organizational commitment, and psychological well-being, while exhibiting a reduced likelihood of adverse outcomes including burnout (Caesens et al., 2014; Zhang et al., 2018). In contrast, low work engagement leads to negative consequences: it reduces work motivation, induces perfunctory work attitudes, and impairs task performance (Coetzee and van Dyk, 2018; Ferreira et al., 2019). For kindergarten teachers, low work engagement may manifest as reduced enthusiasm for interacting with children and diminished responsiveness to their needs, ultimately increasing the probability of turnover intention (Ishibashi et al., 2022; Park and Johnson, 2019).

A growing body of empirical evidence confirms the inverse relationship between adverse workplace gossip and employee work engagement. As a detrimental interpersonal conduct, negative workplace gossip disrupts the psychological state of employees and the quality of the work environment, thereby eroding their work engagement (Cheng et al., 2023; He et al., 2025). From the perspective of conservation of resources theory, individuals strive to acquire, retain, and protect valuable personal resources (e.g., social support, self-esteem, work enthusiasm), and negative workplace gossip directly depletes these critical resources (Hobfoll, 1989; Shehzadi and Khan, 2024). From the lens of conservation of resources theory, individuals make deliberate efforts to obtain, preserve, and safeguard valuable personal resources (e.g., social backing, self-worth, and professional zeal), and negative workplace gossip directly drains these pivotal resources (Cheng et al., 2023; Ullah et al., 2021). This resource drain leaves teachers with insufficient motivation to invest in their work, leading to reduced vigor, diminished dedication, and impaired absorption—key manifestations of low work engagement (Coetzee and van Dyk, 2018; Gan et al., 2023).

From the perspective of social exchange theory, the relationship between employees and their respective organizations is governed by the norm of reciprocity: employees invest time, energy, and enthusiasm (i.e., high work engagement) in exchange for positive returns such as organizational care, colleague respect, and a supportive work environment (Blau, 1964; Cropanzano and Mitchell, 2005). However, negative workplace gossip destroys the positive social exchange between employees and colleagues, and even between employees and the organization. When kindergarten teachers perceive that their efforts and professional integrity are met with unfounded gossip rather than recognition, they feel that the reciprocal exchange relationship is violated (Qi et al., 2022; Tricahyadinata et al., 2020). This perceived imbalance reduces their willingness to invest in work, leading to decreased work engagement (Adil et al., 2020; Shehzadi and Khan, 2024).

Low work engagement further contributes to kindergarten teachers’ turnover intention. When teachers lack sufficient work engagement, they fail to experience meaning and fulfillment from their work, and may even develop a sense of boredom, emotional fatigue, and powerlessness (Parray et al., 2023; Zhang et al., 2018). Kindergarten teaching requires sustained emotional investment and patience; long-term low engagement makes teachers physically and mentally exhausted, prompting them to seek escape from the current work environment (Coetzee and van Dyk, 2018; Park and Johnson, 2019). From the conservation of resources theory perspective, low work engagement indicates severe depletion of work-related resources, and leaving the organization becomes a strategy to prevent further resource erosion (Cheng et al., 2023; Hobfoll et al., 2017). Empirical studies have validated this chain of influence: Coetzee and van Dyk (2018) found that negative workplace behaviors (e.g., bullying, gossip) reduce employees’ work engagement, which subsequently amplifies employees’ intent to leave; Zhang et al. (2018) demonstrated in an investigation of Chinese township health inspectors that work engagement exerts a direct inverse impact on turnover intention (*β* = −0.13, *p* < 0.001). Similarly, in educational settings, teachers’ low levels of work engagement emerge as a primary antecedent of their turnover intention (Rusu and Colomeischi, 2020).

By synthesizing the preceding theoretical insights and empirical findings, adverse workplace gossip is associated with diminished work engagement among kindergarten teachers, which may be explained by the erosion of their psychological resources and the disruption of social exchange dynamics. In turn, this diminished engagement is linked to heightened turnover intention in this cross-sectional context. Accordingly, work engagement functions as a mediating mechanism in the association between negative workplace gossip and kindergarten teachers’ turnover intention. Consequently, the second hypothesis of this research is put forward as follows:

*H2: Work engagement serves as a mediating mechanism in the association between negative workplace gossip and turnover intention among kindergarten teachers.*

## The present study

**Figure 1:**
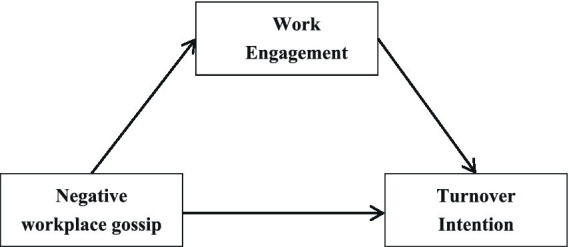
Potential moderated-mediation framework.

Based on conservation of resources theory (Hobfoll, 1989), this research specifically and explicitly puts forward a moderated-mediation model (Figure 1) to illuminate the dynamic factors that shape turnover intention trajectories within the Chinese kindergarten teaching workforce. Specifically, this research focuses on how negative workplace gossip displays a connection with turnover intention, with an emphasis on exploring the underlying mechanism. The results of the present study are anticipated to offer theoretical implications for kindergarten administrators to alleviate and restrain negative workplace gossip, and to provide practical insights for designing intervention strategies that effectively alleviate teachers’ turnover intention.

## Methods

### Participants and procedure

Questionnaire data were gathered through structured surveys, a validated approach to systematically measuring preschool teachers’ subjective constructs through standardized items, which provides a robust empirical basis for large-scale multivariate analysis and theoretical model validation (Dillman et al., 2014). The study protocol was approved by the Ethics Committee of Quzhou Vocational and Technical College (Approval No.: 2025011314). The full approval number is 2025011314 and implemented in strict compliance with the Declaration of Helsinki (World Medical Association, 2013), protecting respondents’ rights to informed consent, privacy, and voluntary participation. A cross-sectional design with convenience sampling was adopted. Self-administered electronic questionnaires were distributed to in-service preschool teachers nationwide via Wenjuanxing (a leading Chinese online survey platform) from April 11 to May 12, 2025, focusing on three core constructs: negative workplace gossip, work engagement, and intent to leave. Data were collected using the Negative Workplace Gossip Scale, Intent to Leave Scale, Work Engagement Scale, and key demographic variables. For recruitment, questionnaire links were shared with cooperating preschool principals and teachers via WeChat, DingTalk, and QQ (mainstream Chinese social platforms), who forwarded them to their work teams with emphasis on self-determined participation. At the outset of the survey, an informed consent disclosure was presented on the initial page; only participants who clicked “Agree to Participate” (equivalent to written consent) accessed the formal survey. To determine statistical power, G*Power 3.1.9.7 (Faul et al., 2007) was utilized to calculate the minimal required sample size for the proposed multiple regression analyses. The analysis was configured with 2 predictor variables, a small effect size (*f^2^* = 0.075), a power level of 0.95, and a significance level of *α* = 0.05. Results revealed that at least 209 participants were needed. In total, 1,418 preschool teachers completed the questionnaire. Rigorous data screening was performed to ensure quality: patterned responses and abnormal completion times (<3 min or >12 min, indicative of inattentive participation) were excluded. Ultimately, 1,306 valid responses were retained for statistical analysis, with an effective response rate of 92.10%. Table 1 provides a complete summary of the demographic profiles included in the study.

**Table 1:** Demographic profile of the study sample (*n* = 1,306).

Variable	Categorical coding	Categorical variable	Count (*n*)	Proportion (%)
Gender	1	Male	33	2.5
	2	Female	1,273	97.5
Marital status	1	Unmarried	508	38.9
	2	Married	798	61.1
Age	1	18–20	73	5.6
	2	21–30	626	47.9
	3	31–40	371	28.4
	4	41–50	194	14.9
	5	51+	42	3.2
Education level	1	High school diploma	66	5.1
	2	Associate degree	568	43.5
	3	Bachelor’s degree	654	50.1
	4	Master’s degree	18	1.4

## Measures

### Negative workplace gossip

To evaluate individuals’ personal perceptions of negative workplace gossip, this research made use of the Perceived Negative Workplace Gossip Scale developed by Chandra and Robinson (2009). This 3-item tool measures how frequently employees feel they are the target of harmful gossip at work, with a sample item being: “Over the past six months, colleagues or supervisors have spread harmful information about me in the workplace.” Participants responded to each item using a 5-point Likert scale, with response options ranging from 1 (signifying *strong disagreement*) to 5 (signifying *strong agreement*). An average composite score was estimated for each member of the sample, where higher scores indicate a higher perceived level of exposure to negative workplace gossip. Previous studies have confirmed the reliability and validity of this instrument within Chinese organizational settings (e.g., Feng et al., 2025), supporting its cross-cultural applicability. In the present study, the scale exhibited robust internal consistency, with a Cronbach’s alpha coefficient of 0.942.

### Intent to leave scale

Turnover intention was assessed via the Intent to Leave Scale, which was initially created by Scott et al. (1999). This 4-item self-report instrument includes a sample item: “I am inclined to seek a more ideal position instead of staying in my current job.” All items were rated on a 5-point Likert scale, with anchors from 1 (*strongly disagree*) to 5 (*strongly agree*). The scale was scored by computing a composite mean, with higher scores corresponding to stronger employee intentions to quit their present employment. The Chinese version of this scale was subsequently validated and applied in prior empirical research by Feng et al. (2025). In the present work, the scale displayed solid internal reliability, evidenced by a Cronbach’s *α* coefficient of 0.873.

### Work engagement scale

The Ability to Focus Scale, initially developed by Mayer and Gavin (2005) to gauge employees’ perceived psychological safety and their capacity to allocate time and energy to work tasks that drive organizational effectiveness, was adapted and translated into a Chinese version by Li and Yan (2007). This 6-item self-report measure includes the sample item, “My work environment allows me to concentrate on my own work,” and employs a 7-point Likert scale, with responses ranging from 1 (*strongly disagree*) to 7 (*strongly agre*). Four items (items 2–5) are reverse-coded prior to scoring, and a total score is computed such that higher values reflect greater levels of work focus. The Chinese version of this scale was subsequently validated and applied in prior empirical research by Li and Yan (2007). Within this research, the measure showed satisfactory internal reliability, producing a Cronbach’s *α* value of 0.867.

### Control variables

To avoid interference from irrelevant variables, this study controlled demographic variables based on prior research. Demographic characteristics are closely related to the core variables in the current research (negative workplace gossip, turnover intention, and work engagement). We selected age, gender, marital status, and education level as control variables in this study. Younger, male, and unmarried teachers scored higher on negative workplace gossip and turnover intention than their counterparts; higher education level was associated with stronger turnover intention (Feng et al., 2025). Work engagement was positively correlated with age (Gan et al., 2023). Male teachers showed a slightly greater mean work engagement than female teachers, married teachers scored higher than unmarried ones, and work engagement differed significantly across age groups (Sharma and Rajput, 2021). These variables were controlled in subsequent data analysis to eliminate their confounding effects on the research conclusions.

## Statistical analysis

All statistical analyses were performed using IBM SPSS Statistics 29.0 and Amos 31.0, adhering to established protocols for organizational behavior research (Hair et al., 2009). First, common method variance (CMV)—a key concern in self-report surveys—was assessed via single-factor confirmatory factor analysis (CFA; Harris and Mossholder, 1996), consistent with the study’ s SEM framework. All items measuring core constructs were restricted to a single latent factor in Amos 31.0; poor model fit indicated minimal CMV impact on result validity (Podsakoff et al., 2012; Hair et al., 2009). Next, descriptive statistics (means and standard deviations), Pearson correlation analyses, and one-way ANOVA tests were performed to characterize the variables, examine the associations between adverse workplace gossip, work engagement, and turnover intention, and explore demographic variations (age, gender, marital status, and education level). Confirmatory factor analysis (CFA) was then implemented in Amos 31.0 (within the SEM framework) to validate the measurement model, evaluating construct reliability (Cronbach’s α, composite reliability [CR]), convergent validity (average variance extracted [AVE]), and discriminant validity (Fornell and Larcker, 1981; Hair et al., 2009). Finally, the hypothesized partial mediation model (with work engagement mediating the link between adverse workplace gossip and turnover intention) was tested using the SPSS PROCESS macro (Model 4, Version 4.1; Hayes, 2013), with 5,000 bias-corrected bootstraps to compute 95% confidence intervals (CIs); effects were deemed statistically significant if the 95% CIs did not contain zero (Hayes and Preacher, 2013). Demographic factors were incorporated as control variables in all analyses to mitigate confounding effects.

## Results

### Preliminary analyses

A preliminary one-way multivariate analysis of variance (MANOVA) was performed on a sample of 1,306 kindergarten teachers to explore demographic variations in negative workplace gossip, work engagement, and turnover intention. The mean score for negative workplace gossip was 1.88 ± 0.97 (scale 1–5), with approximately 79.8% of teachers scoring below the theoretical midpoint value of 3.00, pointing to the fact that negative workplace gossip is not a pervasive concern among this group. Univariate ANOVA and independent samples t-tests revealed significant differences in negative workplace gossip scores across age (*p* < 0.001), gender (*t* = 3.98, *p* < 0.001), marital status (*t* = 2.56, *p* < 0.05), and education level (*F* = 5.32, *p* < 0.001). To be specific, early-career teachers aged 21–30, male teachers, unmarried staff, and those with a master’s degree reported higher levels of negative workplace gossip. Work engagement obtained a total mean value 5.21 ± 1.26 (scale 1–7), reflecting generally high levels of engagement among participants. Significant demographic differences were observed in work engagement scores across age (*F* = 24.13, *p* < 0.001), gender (*t* = −2.72, *p* < 0.01), marital status (*t* = −7.85, *p* < 0.001), and education level (*F* = 3.49, *p* < 0.05), with older teachers (51 + years), married teachers, and those with a high school diploma demonstrating higher work engagement. Turnover intention had an overall mean score of 2.60 ± 0.97 (scale 1–5), with approximately 56.7% of teachers scoring below the theoretical median of 3.00, suggesting that most teachers had no strong intention to leave their current positions. ANOVA results also showed significant demographic differences in turnover intention scores across age (*F* = 69.24, *p* < 0.001), gender (*t* = 2.74, *p* < 0.01), marital status (*t* = 13.26, *p* < 0.001), and education level (*F* = 7.46, *p* < 0.001). In particular, younger teachers (18–20 years), male teachers, unmarried staff, and those with a master’s degree reported higher turnover intentions, whereas older teachers (51 + years), female teachers, married staff, and those with a high school diploma reported lower intentions (Table 2).

**Table 2:** MANOVA results for study variables by demographics (*n* = 1,306).

Variables	Negative workplace gossip		Work engagement		Turnover intention	
	M (SD)	F/t	M (SD)	F/t	M (SD)	F/t
Age						
18–20	1.77 (0.85)	5.90***	4.83 (1.20)	24.13***	3.49 (0.76)	69.24***
21–30	2.00 (0.99)		4.92 (1.25)		2.95 (1.07)	
31–40	1.80 (0.96)		5.46 (1.18)		2.24 (0.94)	
41–50	1.74 (0.98)		5.70 (1.21)		2.05 (0.91)	
51+	1.52 (0.64)		5.75 (1.24)		1.77 (0.77)	
Gender						
Male	2.54 (1.20)	3.98***	4.62 (1.11)	−2.72**	3.11 (1.17)	2.74**
Female	1.86 (0.96)		5.22 (1.26)		2.59 (1.08)	
Marital status						
Unmarried	1.96 (0.93)	2.56*	4.87 (1.18)	−7.85***	3.07 (1.02)	13.26***
Married	1.82 (0.99)		5.42 (1.27)		2.31 (1.02)	
Education level						
High school diploma	1.63 (0.97)	5.32***	5.61 (1.31)	3.49*	2.20 (1.04)	7.46***
Associate degree	1.79 (0.90)		5.25 (1.25)		2.73 (1.10)	
Bachelor’s degree	1.97 (1.02)		5.15 (1.26)		2.52 (1.05)	
Master’s degree	2.13 (1.04)		4.82 (1.11)		2.89 (1.18)	

M, mean. SD, standard deviation. *p < 0.05, **p < 0.01, and ***p < 0.001.

### Latent variable measurement models

Table 3 displays the comparative fit indices for a sequence of nested confirmatory factor analysis (CFA) models, which were evaluated to examine the discriminant validity of the investigation’s focal constructs. The single-factor model, in which all items were loaded onto one latent variable (combining negative workplace gossip, work engagement, and turnover intention), showed extremely suboptimal fit to the data (χ^2^/df = 65.428, TLI = 0.577, CFI = 0.648, RMSEA = 0.222), failing to satisfy any of the recommended fit criteria (Hair et al., 2011). The two-factor model, which arbitrarily merged two of the three constructs into one latent factor while retaining the third as a separate factor, also demonstrated unsatisfactory fit (χ^2^/df = 48.995, TLI = 0.685, CFI = 0.742, RMSEA = 0.192), with all indices falling well below acceptable thresholds. In contrast, the three-factor model, which treated NWG, WE, and TI as distinct latent factors, demonstrated superior fit to the data (χ^2^/df = 2.488, TLI = 0.990, CFI = 0.994, RMSEA = 0.034). All fit indices for this model surpassed the widely accepted benchmarks (e.g., CFI > 0.90, RMSEA < 0.08; Ferreira-Valente et al., 2016), signifying that the three-factor structure effectively captured the uniqueness of the key constructs. These findings additionally bolster the discriminant validity of the measurement framework and establish a robust basis for examining the hypothesized mediational pathways.

**Table 3:** Goodness-of-fit statistics for competing CFA models.

Model	χ^2^	χ^2^/df	TLI	IFI	NFI	GFI	CFI	RMSEA
One-factor model Combining NWG, WE and TI	4252.819	65.428	0.577	0.648	0.645	0.590	0.648	0.222
Two-factor model Combining NWG, WE and TI	3135.709	48.995	0.685	0.742	0.738	0.660	0.742	0.192
Three-factor model Combining NWG, WE and TI	111.961	2.488	0.990	0.994	0.991	0.987	0.994	0.034

NWG, negative workplace gossip; WE, work engagement; TI, turnover intention.

As presented in Table 4, reliability and validity assessments for the measurement scales yielded favorable results. Composite reliability (CR) values for the core constructs were 0.944 (negative workplace gossip), 0.877 (turnover intention), and 0.875 (work engagement), all surpassing the 0.7 cutoff (Hair et al., 2019), which confirms robust internal consistency across scale items. For convergent validity, the average variance extracted (AVE) values stood at 0.849 (negative workplace gossip), 0.643 (turnover intention), and 0.545 (work engagement), all meeting the 0.5 minimum criterion (Hair et al., 2019), indicating that each construct adequately captured the variance of its respective items.

**Table 4:** Descriptive statistics, reliability, and bivariate correlations among all variables (*n* = 1,306).

Variables	α	CR	AVE	1	2	3	4	5	6	7
1. Age				1						
2. Gender				0.003	1					
3. Marital status				0.62***	0.02	1				
4. Education level				−0.01	−0.11***	0.09***	1			
5. Negative workplace gossip	0.942	0.944	0.849	−0.10***	−0.11***	−0.07**	0.11***	(**0.921**)		
6. Turnover intention.	0.873	0.877	0.643	−0.40***	−0.08**	−0.35***	−0.02	0.39***	(**0.802**)	
7. Work engagement	0.867	0.875	0.545	0.25***	0.08**	0.21***	−0.08**	−0.63***	−0.59***	(**0.738**)
M (SD)				2.62 (0.92)	1.97 (0.16)	1.61 (0.49)	2.48 (0.62)	1.88 (0.97)	2.60 (1.09)	5.21 (1.26)

The bold diagonal entries represent the square roots of the average variance extracted (AVE) values. M, mean. SD, standard deviation. *p < 0.05, **p < 0.01, and ***p < 0.001.

For discriminant validity, the square roots of the AVE values (presented as bold diagonal entries in Table 4: 0.921 for negative workplace gossip, 0.802 for turnover intention, 0.738 for work engagement) exceeded all correlation coefficients between the target construct and other constructs within the model. This finding satisfies the Fornell-Larcker criterion (Fornell and Larcker, 1981), confirming that each construct is empirically distinguishable from the others.

Table 4 also presents descriptive statistics (means and standard deviations) and bivariate correlations among all variables (*n* = 1,306). In line with theoretical expectations, negative workplace gossip was positively associated with turnover intention (*r* = 0.39, *p* < 0.001) and negatively associated with work engagement (*r* = −0.63, *p* < 0.001). Turnover intention also displayed a strong negative association with work engagement (*r* = −0.59, *p* < 0.001). Additionally, demographic factors showed notable associations: age was positively associated with work engagement (*r* = 0.25, *p* < 0.001) and negatively associated with turnover intention (*r* = −0.40, *p* < 0.001), while marital status was positively associated with work engagement (*r* = 0.21, *p* < 0.001) and negatively associated with turnover intention (*r* = −0.35, *p* < 0.001).

### Testing the mediation model

**Figure 2:**
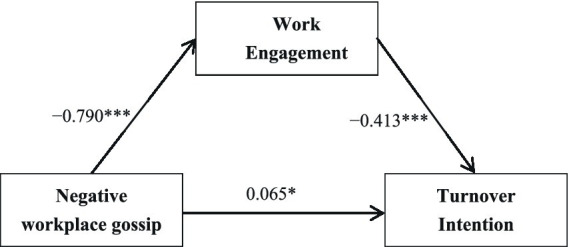
Mediation pathways of negative workplace gossip, turnover intention, work engagement. *p < 0.05 and ***p < 0.001.

Hayes’s (2013) PROCESS macro (Model 4) was utilized to investigate the mediating function of work engagement in the association between negative workplace gossip and turnover intention, while adjusting for age, gender, marital status, and educational attainment. The total effect of negative workplace gossip on turnover intention was significant and positive (*β* = 0.391, *p* < 0.001), indicating that higher levels of negative workplace gossip were linked to greater turnover intention among kindergarten educators. According to the results in Table 5, the first-stage regression showed that negative workplace gossip was a significant negative predictor of work engagement (*β* = −0.790, *p* < 0.001, 95% CI [−0.844, −0.735]). In the second-stage regression, both negative workplace gossip (*β* = 0.065, *p* < 0.05, 95% CI [0.005, 0.125]) and work engagement (*β* = −0.413, *p* < 0.001, 95% CI [−0.460, −0.366]) emerged as significant predictors of turnover intention. The model accounted for 43.5% of the variance in work engagement (*F* = 199.829, *p* < 0.001) and 42.9% of the variance in turnover intention (*F* = 162.644, *p* < 0.001). Demographic control variables also exerted significant effects: age and marital status were positive predictors of work engagement, whereas age, marital status, and educational attainment were negative predictors of turnover intention. When work engagement was added to the model, the direct influence of negative workplace gossip on turnover intention remained statistically significant. The indirect effect via work engagement (calculated as −0.790 × −0.413 = 0.326) was also significant, with its 95% confidence interval not containing zero. This confirms that work engagement partially mediates the association between negative workplace gossip and turnover intention, accounting for a substantial portion of the total effect (Figure 2).

**Table 5:** The mediating role of work engagement in the association between negative workplace gossip and turnover intention.

Predictors	Work engagement			Turnover intention		
	β	SE	95%CI	β	SE	95% CI
Constant	5.873***	0.379	[5.130, 6.616]	6.412***	0.357	[5.712, 7.112]
Age	0.181***	0.037	[0.109, 0.254]	−0.255***	0.032	[−0.318, −0.192]
Gender	0.036	0.169	[−0.297, 0.368]	−0.253	0.147	[−0.541, 0.035]
Marital status	0.231***	0.069	[0.095, 0.367]	−0.221***	0.060	[−0.339, −0.102]
Education level	−0.040	0.043	[−0.125, 0.046]	−0.103**	0.038	[−0.177–0.029]
Negative workplace gossip	−0.790***	0.028	[−0.844, −0.735]	0.065*	0.031	[0.005, 0.125]
Work engagement				−0.413***	0.024	[−0.460, −0.366]
*R^2^*	0.435			0.429		
*F*	199.829***			162.644***		

A total of 5,000 bootstrap resamples were utilized. *p < 0.05, **p < 0.01, ***p < 0.001.

## Discussion

The present study examined the association between negative workplace gossip and turnover intention within a group of Chinese kindergarten teachers, along with the potential mediating function of work engagement, grounded in conservation of resources (COR) theory. Data were collected at a single time point to describe the relationships among the variables, and causal inferences cannot be drawn from the study design. Overall, the results supported both proposed hypotheses: negative workplace gossip was positively linked to turnover intention, and work engagement partially mediated this linkage. These findings deepen the understanding of the psychological mechanism by which negative interpersonal behaviors may influence early childhood teachers’ career retention and provide empirical evidence for reducing teacher turnover in the preschool education sector.

First, the empirical data uncovered a significant positive association between negative workplace gossip and turnover intention, thereby supporting Hypothesis 1. Such a relationship pattern aligns with prior empirical investigations across diverse occupational settings, suggesting that perceived negative workplace gossip is tied to elevated levels of turnover intention (Anjarwati and Larasati, 2025; Coetzee and van Dyk, 2018). Although existing studies have well documented ECE teacher attrition and its conventional predictors (e.g., heavy workload, low salary, institutional pressure), few clarify how negative workplace gossip specifically affects Chinese kindergarten teachers (Feng et al., 2025; Xu et al., 2026). Unlike Western teachers with independent work boundaries and sufficient personal coping resources, Chinese kindergarten teachers work in closed, high-collaborative environments shaped by collectivist norms that prioritize interpersonal harmony (He et al., 2023; Xu et al., 2026). For the sampled teachers, targeted negative gossip behaviors, including character assassination, misleading information, unverified rumors, and unfavorable professional comments, act as severe interpersonal stressors. These harmful acts trigger workplace conflict and victimization, induce psychological stress and emotional exhaustion, reduce job satisfaction and organizational belonging, and ultimately drive turnover intention (He and Wei, 2022; Wang et al., 2022). The total effect of negative workplace gossip on turnover intention was significant and positive (*β* = 0.391, *p* < 0.001). After controlling for work engagement, its direct effect remained significant (*β* = 0.065, *p* < 0.05), which is consistent with the core prediction of Hypothesis 1. Negative workplace gossip operates as a detrimental interpersonal stressor that undermines relational trust and psychological well-being (He et al., 2023; Shehzadi and Khan, 2024), a dynamic that is especially consequential for kindergarten teachers, who depend on collaborative, positive interpersonal interactions to meet the intensive emotional labor requirements of their role. From the perspective of COR theory, the findings suggest that exposure to negative gossip may deplete emotional and social resources—critical for maintaining occupational commitment—thus potentially strengthening teachers’ inclination to withdraw from the current work setting (Hobfoll et al., 2017; Rasool et al., 2021). Notably, the present result contrasts with studies where workplace bullying or incivility failed to show significant effects on turnover intention (Anjarwati and Larasati, 2025), highlighting that negative workplace gossip may be a more salient interpersonal stressor for kindergarten teachers due to the profession’s emphasis on trust and collegial support. Importantly, the observed association is consistent with the hypothesized directional mechanism and extends previous findings to the Chinese kindergarten teacher population, reinforcing that negative workplace gossip is a key correlate of turnover intention in this specific occupational group (He et al., 2023).

Second, the study findings validated the mediating function of work engagement in the association between negative workplace gossip and turnover intention (Hypothesis 2), which is consistent with broader research on workplace stressors and employee outcomes (Coetzee and van Dyk, 2018; Gadi and Kee, 2020). In line with prior investigations, negative workplace gossip was negatively linked to work engagement (Cheng et al., 2023; He et al., 2025), and work engagement in turn was negatively predictive of turnover intention (Asghar et al., 2021; Zhang et al., 2018). The study’s mediation analysis further validated this mediating mechanism: negative workplace gossip served as a significant negative predictor of work engagement (*β* = −0.790, *p* < 0.001), and work engagement was a significant negative predictor of turnover intention (*β* = −0.413, *p* < 0.001), with the indirect pathway of negative workplace gossip on turnover intention via work engagement being significant (indirect effect = 0.326, 95% CI excluding zero). Within the COR framework, the data indicate that negative workplace gossip may deplete key psychological resources including emotional energy, social support, and a sense of respect (Hobfoll et al., 2017), thereby reducing teachers’ vigor, dedication, and absorption—the core dimensions of work engagement (Bakker and Demerouti, 2007).

For kindergarten teachers, reduced work engagement, as reflected in the study data, manifests as diminished patience with children, lack of enthusiasm for professional development, and difficulty concentrating on care tasks, which in turn may erode occupational commitment and strengthen turnover intention (Park and Johnson, 2019; Ma et al., 2021). This mediating pathway is consistent with research showing that work engagement functions as a crucial buffer between adverse workplace experiences (e.g., bullying, toxic environments) and turnover intention (Coetzee and van Dyk, 2018; Rasool et al., 2021). The partially mediated model suggests that negative workplace gossip is linked to turnover intention both directly—stemming from immediate psychological discomfort and social exclusion—and indirectly through potential resource depletion and reduced work engagement. This dual associative pathway clarifies the psychological mechanism linking negative interpersonal experiences to occupational withdrawal among Chinese kindergarten teachers, complementing prior research on mediating variables such as ego depletion (He and Wei, 2022) and psychological contract breach (He et al., 2023) in workplace gossip settings.

Notably, the present investigation centered on Chinese kindergarten teachers—an occupational group facing unique challenges such as high workloads, resource constraints, and a strong emphasis on interpersonal harmony (He et al., 2023)—highlighting the contextual relevance of the findings. In collectivist cultural contexts, interpersonal relationships carry heightened significance (He et al., 2023), making negative gossip particularly damaging to psychological well-being and work engagement. Unlike Western contexts where individual autonomy and diverse coping resources may mitigate the impact of interpersonal stressors (Mazzetti et al., 2021), Chinese kindergarten teachers’ heavy reliance on collegial support and organizational belonging amplifies the resource-depleting effects of negative gossip (Anjarwati and Larasati, 2025; He et al., 2023). In collectivist settings, workplace relationships are central to professional identity and well-being, so gossip that undermines trust and social standing can erode the psychological resourcesessential for preserving work engagement (He et al., 2023; Rasool et al., 2021). This contrasts with more individualistic contexts, where employees may draw on personal agency to buffer such negative experiences, whereas Chinese kindergarten teachers’ occupational identity is deeply intertwined with their relational standing within the school community (Anjarwati and Larasati, 2025; He et al., 2023; Mazzetti et al., 2021). Additionally, the high-density, interactive nature of kindergarten work environments accelerates gossip spread and prolongs its emotional impact (He and Wei, 2022), further reinforcing the mediating function of work engagement as a resource-dependent psychological state in the observed associations. These contextual factors explain why the observed relationships are robust in the current sample, extending the generalizability of COR theory to early childhood education settings in collectivist cultures (Mazzetti et al., 2021).

The results of this empirical work also correspond with previous investigations on professional identity and job satisfaction, which have been identified as key correlates of turnover intention among public sector employees (Zhang et al., 2018). Although the present inquiry centered on work engagement as a mediating variable, the results indirectly support the broader proposition that positive psychological states (e.g., engagement, satisfaction) may mitigate the negative effects of workplace stressors (Rasool et al., 2021). In the context of kindergarten teachings, work engagement serves as a critical psychological resource that may offset the resource loss caused by negative gossip, consistent with COR theory’s core principle that resource enhancement buffers against stress (Hobfoll et al., 2017). This is consistent with longitudinal investigations demonstrating that work engagement fosters retention by sustaining occupational commitment over time (Trépanier et al., 2014), though the study design prevents confirmation of such long-term effects.

In summary, this empirical investigation validates that negative workplace gossip is a notable predictor of heightened turnover intention among Chinese kindergarten teachers, with work engagement functioning as a partial mediating variable. The results capture the interconnections among variables at a single time point, and causal inferences cannot be drawn; future longitudinal inquiries are required to confirm the proposed causal pathways. These outcomes integrate and advance existing literature on workplace gossip, work engagement, and turnover intention, while emphasizing the distinct contextual dynamics of early childhood education in China. The findings underscore the significance of addressing negative interpersonal behaviors and enhancing work engagement to reduce teacher turnover, delivering actionable insights for educational administrators and policymakers.

### Theoretical contributions

This study broadens the explanatory reach of Conservation of Resources (COR) theory by corroborating its utility in unpacking the influence of negative workplace gossip—an often-overlooked interpersonal stressor—on kindergarten teachers’ turnover intention. As a core theoretical framework in organizational behavior, COR theory postulates that individuals inherently strive to safeguard and accumulate valuable resources, and exposure to stressors that deplete these resources will trigger withdrawal behaviors to prevent additional resource loss (Hobfoll et al., 2017). Yet, prior COR theory research in organizational and educational contexts has primarily focused on traditional task-related stressors, including excessive workload, role conflict, and job insecurity (Rasool et al., 2021), with limited attention to the unique interpersonal dynamics that characterize care-oriented professions ECE. This investigation demonstrates that negative workplace gossip acts as a distinct resource-depleting stressor, as it erodes key psychological resources—specifically work engagement—and subsequently prompts turnover intention among kindergarten teachers. This contribution expands COR theory’s application beyond task-focused stressors to interpersonal stressors, which are particularly salient in ECE settings where emotional labor, collaborative relationships, and relational trust are foundational to both teacher well-being and job performance (He et al., 2023; Cheng et al., 2023).

This study addresses a critical gap in the extant literature by establishing work engagement as a pivotal mediating pathway that connects negative workplace gossip to turnover intention, offering greater nuance beyond the individual-level cognitive and emotional mediators focused on in prior research (e.g., ego depletion, He and Wei, 2022; psychological contract breach, He et al., 2023). As a multi-dimensional construct encompassing vigor, dedication, and absorption (Bakker, 2014), work engagement directly reflects teachers’ proactive investment in their work and their ability to sustain psychological resources amid workplace stress. Importantly, work engagement is malleable to organizational intervention—unlike many individual-level constructs that are difficult to modify—making it highly contextually relevant to ECE settings seeking to improve teacher retention. Our findings align with recent research that highlights work engagement’s critical mediating role between workplace stressors and turnover (Coetzee and van Dyk, 2018; Shehzadi and Khan, 2024), while further emphasizing its unique significance for ECE, where high levels of teacher work engagement are not only essential for reducing turnover but also for ensuring high-quality child care and early childhood development (Rusu and Colomeischi, 2020).

By focusing on Chinese kindergarten teachers—a sample embedded in a collectivist cultural context—this study effectively addresses the long-standing cultural gap in workplace gossip research, which has historically been dominated by Western, individualistic samples. Collectivist cultures, such as Chinese society, place strong emphasis on interpersonal harmony, social connectedness, and group cohesion, which makes interpersonal stressors like negative workplace gossip particularly damaging to individuals’ psychological well-being and organizational attachment. Our results demonstrate that these collectivist norms amplify the harmful impacts 0f negative workplace gossip: beyond depleting individual psychological resources, gossip violates the collective norms of mutual respect and harmony that underpin social interactions in such contexts (He et al., 2023; Cheng et al., 2023). This cross-cultural extension challenges the generalizability of Western-based findings, which often prioritize individual autonomy and downplay the impact of interpersonal conflict on withdrawal behaviors (Mazzetti et al., 2021), and ultimately provides a more comprehensive, cross-cultural framework for understanding how cultural norms shape the impact of interpersonal stressors in care-oriented professions like ECE.

### Implications for practice

This study demonstrates that negative workplace gossip positively predicts turnover intention among Chinese kindergarten educators, with work engagement acting as a key mediator. Importantly, the observed correlations do not indicate causality. Based on these findings, this section presents concise, actionable, high-impact strategies at the institutional, individual, and societal levels to mitigate the detrimental effects of negative gossip, enhance teacher work engagement, and stabilize the early childhood education workforce.

First, kindergartens should regulate workplace communication and foster a supportive climate to constrain negative gossip at its source. Schools should establish clear communication and anti-gossip policies that classify the spread of unsubstantiated rumors regarding colleagues’ work performance, private lives, and professional ethics as behavioral misconduct (Jiang et al., 2020). Timely internal rumor clarification through official notices and team meetings can limit the proliferation of false information. Administrators should model respectful and transparent communication, avoid gossip, and intervene promptly and equitably when issues arise, including one-on-one consultations and standardized disciplinary reminders where appropriate. These practices enhance teachers’ interpersonal security, reduce gossip-related workplace insecurity, and sustain stable work engagement.

Second, targeted organizational support can strengthen work engagement and buffer gossip-induced stress, especially for vulnerable teachers aged 21–30 who reported greater gossip exposure in this study. Kindergartens can implement mentorship programs to provide new and at-risk teachers with ongoing emotional and instructional support. Schools should also offer accessible professional development resources, including pedagogical training, research guidance, and career counseling, to improve teacher competence and occupational fulfillment (Hall, 2019). Additionally, involving teachers in institutional and curricular decision-making and delivering timely recognition and rewards for strong performance enhances job meaningfulness and professional belonging. Such support mitigates the adverse effects of gossip-related stress and maintains teacher enthusiasm and dedication.

Third, individual teachers should enhance their psychological adaptability to reduce gossip vulnerability. Participation in school-based psychological training can help teachers master emotion-regulation strategies, such as mindfulness and cognitive reappraisal, to alleviate gossip-triggered anxiety, frustration, and self-doubt. Teachers should maintain clear interpersonal boundaries, disengage from workplace gossip, and prioritize teaching quality, child interaction, and professional research (Zhang et al., 2025). Cultivating mindfulness and building trust with supervisors and colleagues further weakens the negative influence of unfounded rumors on work engagement and professional commitment (Green, 2019).

Finally, educational authorities and social institutions should optimize the external occupational environment to reduce relationship-driven turnover intention. Improving kindergarten teachers’ base salaries, social security benefits, and career promotion pathways can alleviate economic and professional anxiety (Ji and Cui, 2021; Panagopoulos et al., 2014). Public advocacy campaigns should elevate teachers’ social status and professional identity by highlighting their developmental and educational contributions, thereby reducing gossip sensitivity. Furthermore, public psychological counseling services and industry peer support platforms can improve teacher satisfaction and organizational loyalty, stabilize the early childhood education talent pool, and support the sector’s high-quality development.

### Limitations and future directions

It is imperative to acknowledge several inherent limitations of the present study, alongside promising avenues for subsequent scholarly inquiry. First, this study utilized a cross-sectional design, which only measures variables at a single time point and therefore cannot establish causal or longitudinal associations among negative workplace gossip, work engagement, and turnover intention; future research could adopt longitudinal or experimental designs to validate the causal direction and temporal stability of the proposed model. Second, all data were gathered via self-administered questionnaires, which may give rise to common method bias; while statistical analyses confirmed minimal bias, future research could incorporate multisource data (e.g., supervisor ratings, peer evaluations, or organizational records) to mitigate subjective reporting bias. Third, this study only explored the mediating function of work engagement, without exploring potential moderating variables that may buffer or strengthen the relationships under investigation; future studies could incorporate personal factors (e.g., psychological capital, emotional intelligence) or contextual factors (e.g., leadership style, organizational climate, social support) as moderators to develop a more comprehensive moderated mediation model. Fourth, the study employed convenience sampling, which may constrain the generalizability of the results; future research could adopt probability sampling across diverse regions, public and private kindergartens, and urban–rural settings to strengthen representativeness. Fifth, this study focused solely on perceived negative workplace gossip as a unidimensional construct; future studies could distinguish between different forms, sources, or functions of gossip (e.g., victim vs. observer perspectives, gossip from colleagues vs. supervisors) to yield a more detailed and nuanced comprehension. Finally, this study was carried out with Chinese kindergarten teachers within a collectivist cultural context; cross-cultural comparative research could be performed to examine whether the associations differ across individualist and collectivist cultures or among different care-oriented professions.

## Conclusion

This study explores the associations between negative workplace gossip, work engagement, and turnover intention among Chinese kindergarten teachers, rooted in the Conservation of Resources (COR) theory. Employing a cross-sectional design, data were gathered from 1,306 practicing kindergarten teachers via questionnaires, and analyzed using IBM SPSS 29.0 and Amos 31.0. The key findings support both proposed hypotheses: negative workplace gossip is positively linked to turnover intention, and work engagement partially mediates the association between negative workplace gossip and turnover intention. Demographically, younger, male, and unmarried teachers, as well as those with higher education levels, report higher negative workplace gossip and turnover intention, while in contrast older and married teachers show higher work engagement. Theoretically, this research extends the application of COR theory to interpersonal stressors in early childhood education (ECE), identifies work engagement as a critical mediating pathway, and enriches cross-cultural research on workplace gossip in collectivist contexts. Practically, the findings provide actionable implications: kindergartens should establish anti-gossip norms and enhance organizational support to improve teachers’ work engagement; educational authorities should optimize the external support system for early childhood education teachers; and individual teachers should strengthen their psychological resilience to alleviate the adverse effects of negative workplace gossip. While recognizing constraints such as the cross-sectional design and self-reported data, this research enhances the comprehension of the psychological mechanisms underlying kindergarten teachers’ turnover intention, offering empirical support for stabilizing the early childhood education workforce and laying a foundation for subsequent research.

## Data Availability

The raw data supporting the conclusions of this article will be made available by the authors, without undue reservation.

## References

[ref1] Adil M. S. Hamid K. B. A. Waqas M. (2020). Impact of perceived organisational support and workplace incivility on work engagement and creative work involvement: a moderating role of creative self-efficacy. Int. J. Manag. Pract. 13, 117–138. doi: 10.1504/ijmp.2020.105671

[ref2] Aggarwal A. Chand P. K. Jhamb D. Mittal A. (2020). Leader–member exchange, work engagement, and psychological withdrawal behavior: the mediating role of psychological empowerment. Front. Psychol. 11:423. doi: 10.3389/fpsyg.2020.00423, 32296361 PMC7136488

[ref3] Akasheh M. Al Hujran O. Malik E. F. Zaki N. (2024). Enhancing the prediction of employee turnover with knowledge graphs and explainable AI. IEEE Access 12, 77041–77053. doi: 10.1109/ACCESS.2024.3404829

[ref4] Anjarwati P. O. Larasati N. (2025). Pengaruh workplace bullying, workplace gossip dan workplace incivility Terhadap turnover intention pada Pekerja gen Z di Kota Surakarta. PENG Jurnal Ekonomi dan Manajemen 3, 811–827. doi: 10.62710/vecs1390

[ref9001] Asghar M. Tayyab M. Gull N. Song Z. Shi R. Tao X. (2021). Polychronicity, work engagement, and turnover intention: The moderating role of perceived organizational support in the hotel industry. J. Hosp. Tour. Manag., 49, 129–139. doi: 10.1016/j.jhtm.2021.09.004

[ref5] Bakker A. B. (2014). Daily fluctuations in work engagement. Eur. Psychol. 19, 227–236. doi: 10.1027/1016-9040/a000160

[ref6] Bakker A. B. Demerouti E. (2007). The job demands-resources model: state of the art. J. Manag. Psychol. 22, 309–328. doi: 10.1108/02683940710733115

[ref7] Bellows L. Bassok D. Markowitz A. J. (2022). Teacher turnover in early childhood education: longitudinal evidence from the universe of publicly funded programs in Louisiana. Educ. Res. 51, 565–574. doi: 10.3102/0013189X221131505

[ref8] Blau P. M. (1964). Exchange and Power in Social Life. New York: John Wiley, 153–160.

[ref9] Brok P. D. Wubbels T. Tartwijk J. V. (2017). Exploring beginning teachers’ attrition in the Netherlands. Teach. Teach. 23, 881–895. doi: 10.1080/13540602.2017.1360859

[ref10] Caesens G. Stinglhamber F. Luypaert G. (2014). The impact of work engagement and workaholism on well-being. Career Dev. Int. 19, 813–835. doi: 10.1108/cdi-09-2013-0114

[ref11] Chandra G. Robinson S. L. (2009). They’re talking about me again: the impact of being the target of gossip on emotional distress and withdrawal Academy of Management Annual Meeting, Chicago, IL, United States.

[ref12] Cheng X. Duan J. Wu W. Lu L. (2023). From the dual-dimensional perspective of employee mindfulness and superior trust, explore the influence mechanism of negative workplace gossip on work engagement. Front. Psych. 14:1287217. doi: 10.3389/fpsyt.2023.1287217, 38076705 PMC10704129

[ref13] Choi J. Y. Horm D. Jeon S. Ryu D. (2019). Do stability of care and teacher–child interaction quality predict child outcomes in early head start? Early Educ. Dev. 30, 337–356. doi: 10.1080/10409289.2018.1546096

[ref14] Coetzee M. van Dyk J. (2018). Workplace bullying and turnover intention: exploring work engagement as a potential mediator. Psychol. Rep. 121, 375–392. doi: 10.1177/0033294117725073, 28812953

[ref15] Cropanzano R. Mitchell M. S. (2005). Social exchange theory: an interdisciplinary review. J. Manag. 31, 874–900. doi: 10.1177/0149206305279602

[ref16] Di H. Li H. Wang Y. (2023). Kindergarten teachers’ quality of work life in China: a National Empirical Study. Int. J. Environ. Res. Public Health 20:4596. doi: 10.3390/ijerph20054596, 36901605 PMC10002027

[ref17] Dillman D. A. Smyth J. D. Christian L. M. (2014). Internet, phone, mail, and Mixed-mode Surveys: The Tailored design Method. 4th Edn Hoboken, NJ, USA: John Wiley & Sons.

[ref18] Doromal J. B. Bassok D. Bellows L. Markowitz A. J. (2022). Hard-to-staff centers: exploring center-level variation in the persistence of child care teacher turnover. Early Child Res. Q. 61, 170–178. doi: 10.1016/j.ecresq.2022.07.007

[ref19] Dunlop R. Scheepers C. B. (2022). The influence of female agentic and communal leadership on work engagement: vigour, dedication and absorption. Manag. Res. Rev. 46, 437–466. doi: 10.1108/mrr-11-2021-0796

[ref20] Durmuş A. Ünal Ö. Türktemiz H. Öztürk Y. E. (2024). The effect of nurses' perceived workplace incivility on their presenteeism and turnover intention: the mediating role of work stress and psychological resilience. Int. Nurs. Rev. 71, 960–968. doi: 10.1111/inr.12950, 38465769 PMC11600495

[ref21] Faul F. Erdfelder E. Lang A. G. Buchner A. (2007). G* power 3: a flexible statistical power analysis program for the social, behavioral, and biomedical sciences. Behav. Res. Methods 39, 175–191. doi: 10.3758/BF03193146, 17695343

[ref22] Fee K. D. (2024). Using worker flows to assess the stability of the early childcare and education workforce, 2010-2022. Federal Reserve Bank Cleveland Community Develop. Rep. doi: 10.26509/frbc-cd-20240119

[ref23] Feng B. Dou G. Zhan X. (2025). Negative workplace gossip and turnover intention among kindergarten teachers: psychological safety as a mediator and organizational identification as a moderator. Front. Psychol. 16:1588482. doi: 10.3389/fpsyg.2025.1588482, 40606906 PMC12218253

[ref24] Ferreira A. I. da Costa Ferreira P. Cooper C. L. Oliveira D. (2019). How daily negative affect and emotional exhaustion correlates with work engagement and presenteeism-constrained productivity. Int. J. Stress. Manag. 26, 261–271. doi: 10.1037/str0000114

[ref25] Ferreira-Valente A. Costa P. Elorduy M. Virumbrales M. Costa M. J. Palésl J. (2016). Psychometric properties of the Spanish version of the Jefferson scale of empathy: making sense of the total score through a second order confirmatory factor analysis. BMC Med. Educ. 16:242. doi: 10.1186/s12909-016-0763-5, 27647296 PMC5028960

[ref26] Fornell C. Larcker D. F. (1981). Evaluating structural equation models with unobservable variables and measurement error. J. Mark. Res. 18, 39–50. doi: 10.1177/002224378101800104

[ref27] Gadi P. D. Kee D. M. H. (2020). Workplace bullying, human resource management practices, and turnover intention: the mediating effect of work engagement: evidence of Nigeria. Am. J. Bus. 36, 62–83. doi: 10.1108/ajb-08-2020-0135

[ref28] Gan S. K.-E. Zeng Y. Wang Z. (2023). Social anxiety mediates workplace incivility and work engagement. Front. Psychol., 14. doi: 10.3389/fpsyg.2023.1320703 PMC10715392 38090173

[ref29] Grant A. A. Jeon L. Buettner C. K. (2019). Relating early childhood teachers’ working conditions and well-being to their turnover intentions. Educ. Psychol. 39, 294–312. doi: 10.1080/01443410.2018.1543856

[ref30] Green C. A. (2019). Workplace incivility. Nurs. Manag. 50, 51–53. doi: 10.1097/01.numa.0000550455.99449.6b, 30601385

[ref31] Hair J. F. Black W. C. Babin B. J. Anderson R. E. (2009). Multivariate data analysis. 7th Edn Pearson. Upper Saddle River, New Jersey, USA: Prentice Hall.

[ref32] Hair J. F. Jr. Black W. C. Babin B. J. Anderson R. E. (2019). Multivariate data analysis. 8th Edn. Boston, Massachusetts, USA: Cengage Learning.

[ref33] Hair J. F. Ringle C. M. Sarstedt M. (2011). PLS-SEM: indeed a silver bullet. J. Mark. Theory Pract. 19, 139–152. doi: 10.2753/mtp1069-6679190202

[ref34] Hall A. B. (2019). Personalized Professional Learning Experiences and Teacher Self-Efficacy for Integrating Technology in K-12 Classrooms [Doctoral Dissertation]. ScholarWorks @ Boise State University.

[ref35] Harris S. G. Mossholder K. W. (1996). The affective implications of perceived congruence with culture dimensions during organizational transformation. J. Manag. 22, 527–547. doi: 10.1177/014920639602200401

[ref36] Hayes A. (2013). Introduction to mediation, moderation, and conditional process analysis. J. Educ. Meas. 51, 335–337. doi: 10.1111/jedm.12050, 40046247

[ref37] Hayes A. F. Preacher K. J. (2013). Statistical mediation analysis with a multicategorical independent variable. Br. J. Math. Stat. Psychol. 67, 451–470. doi: 10.1111/bmsp.12028, 24188158

[ref38] He Q. Chen P. Yang F. Que J. Hu X. (2025). The contest between sensibility and rationality: a study on the influence of negative workplace gossip on work engagement. Nankai Bus. Rev. Int. 16, 385–408. doi: 10.1108/nbri-07-2024-0074

[ref39] He C. Feng T. Xiong J. Hua W. (2023). The relationship between negative workplace gossip and thriving at work among Chinese kindergarten teachers: the roles of psychological contract breach and bianzhi. Front. Psychol. 14:1198316. doi: 10.3389/fpsyg.2023.1198316, 37538995 PMC10394615

[ref40] He C. Wei H. (2022). Negative workplace gossip and turnover intention among Chinese rural preschool teachers: the mediation of ego depletion and the moderation of bianzhi. Front. Psychol. 13:1034203. doi: 10.3389/fpsyg.2022.1034203, 36533038 PMC9755678

[ref41] Hobfoll S. E. (1989). Conservation of resources: a new attempt at conceptualizing stress. Am. Psychol. 44, 513–524. doi: 10.1037/0003-066x.44.3.513, 2648906

[ref42] Hobfoll S. E. Halbesleben J. R. B. Neveu J. Westman M. (2017). Conservation of resources in the organizational context: the reality of resources and their consequences. Annu. Rev. Organ. Psychol. Organ. Behav. 5, 103–128. doi: 10.1146/annurev-orgpsych-032117-104640

[ref43] Hur E. H. Ardeleanu K. Satchell T. W. Jeon L. (2023). Why are they leaving? Understanding associations between early childhood program policies and teacher turnover rates. Child Youth Care Forum 52, 417–440. doi: 10.1007/s10566-022-09693-x

[ref44] Ishibashi S. Tokunaga A. Shirabe S. Yoshida Y. Imamura A. Takahashi K. . (2022). Burnout among kindergarten teachers and associated factors. Medicine 101:e30786. doi: 10.1097/md.0000000000030786, 36197261 PMC9509133

[ref45] Ji D. Cui L. (2021). Relationship between Total rewards perceptions and work engagement among Chinese kindergarten teachers: organizational identification as a mediator. Front. Psychol. 12:648729. doi: 10.3389/fpsyg.2021.648729, 33995206 PMC8116523

[ref46] Jiang M. Gao Q. Zhuang J. (2020). Reciprocal spreading and debunking processes of online misinformation: a new rumor spreading–debunking model with a case study. Phys. A Stat. Mech. Appl. 565:125572. doi: 10.1016/j.physa.2020.125572

[ref47] Lee S. E. Seo J.-K. Macphee M. (2024). Effects of workplace incivility and workload on nurses’ work attitude: the mediating effect of burnout. J. Adv. Nurs. 71:1080. doi: 10.1111/inr.12974, 38661534 PMC11600474

[ref48] Li N. Yan J. (2007). The mechanism of how trust climate impacts on individual performance. Acta Psychol. Sin. 39, 1111–1121. Available online at: https://journal.psych.ac.cn/acps/EN/Y2007/V39/I06/1111

[ref49] Ma L. Zhou F. Liu H. (2021). Relationship between psychological empowerment and the retention intention of kindergarten teachers: a chain intermediary effect analysis. Front. Psychol. 12:601992. doi: 10.3389/fpsyg.2021.601992, 33679521 PMC7928276

[ref50] Mayer R. C. Gavin M. B. (2005). Trust in management and performance: who minds the shop while the employees watch the boss? Acad. Manag. J. 48, 874–888. doi: 10.5465/amj.2005.18803928

[ref51] Mazzetti G. Robledo E. Vignoli M. Topa G. Guglielmi D. Schaufeli W. B. (2021). Work engagement: a meta-analysis using the job demands-resources model. Psychol. Rep. 126, 1069–1107. doi: 10.1177/00332941211051988, 34886729

[ref52] Mims S. U. Scott-Little C. Lower J. K. Cassidy D. J. Hestenes L. L. (2008). Education level and stability as it relates to early childhood classroom quality: a survey of early childhood program directors and teachers. J. Res. Child. Educ. 23, 227–237. doi: 10.1080/02568540809594657

[ref53] Mujtaba B. G. Senathip T. (2020). Workplace mobbing and the role of human resources management. Business Ethics Leadership 4, 17–34. doi: 10.21272/bel.4(1).17-34.2020

[ref54] Panagopoulos N. Anastasiou S. Goloni V. (2014). Professional Burnout And Job Satisfaction among Physical Education Teachers In Greece Zenodo (Geneva: CERN European Organization for Nuclear Research, CERN).

[ref55] Park K. A. Johnson K. (2019). Job satisfaction, work engagement, and turnover intention of CTE health science teachers. Int. J. Res. Vocational Educ. Train. 6, 224–242. doi: 10.13152/ijrvet.6.3.2

[ref56] Parray Z. A. Islam S. U. Shah T. A. (2023). Impact of workplace incivility and emotional exhaustion on job outcomes – a study of the higher education sector. Int. J. Educ. Manag. 37, 1024–1041. doi: 10.1108/ijem-07-2022-0267, 35579975

[ref57] Podsakoff P. M. MacKenzie S. B. Lee J. Y. Podsakoff N. P. (2012). Common method biases in behavioral research: a critical review of the literature and recommended remedies. J. Appl. Psychol. 17, 244–254. doi: 10.1037/a0028031, 14516251

[ref58] Puspitasari A. S. Darwin M. (2021). Effect of work-life balance and welfare level on millennial employee performance through work engagement. Int. J. Sci. Soc. 3, 334–344. doi: 10.54783/ijsoc.v3i1.299

[ref59] Qi L. Chaudhary N. I. Yao K. Mirza F. Khalid R. (2022). The moderating role of transformational leadership on the relationship between deviant workplace behaviors and employee turnover intentions in China. Front. Psychol. 13:1005055. doi: 10.3389/fpsyg.2022.1005055, 36304849 PMC9592725

[ref60] Rasool S. F. Wang M. Tang M. Saeed A. Iqbal J. (2021). How toxic workplace environment effects the employee engagement: the mediating role of organizational support and employee wellbeing. Int. J. Environ. Res. Public Health 18:2294. doi: 10.3390/ijerph18052294, 33652564 PMC7956351

[ref61] Ren X. Yan Z. Zhang Z. Chen J. Tian Y. (2024). Current situation and influencing factors of each turnover of kindergarten teachers–a questionnaire survey. Front. Psychol. 15:1321441. doi: 10.3389/fpsyg.2024.1321441, 38414879 PMC10897977

[ref62] Rusu P. P. Colomeischi A. A. (2020). Positivity ratio and well-being among teachers. The mediating role of work engagement. Front. Psychol. 11:1608. doi: 10.3389/fpsyg.2020.01608, 32793041 PMC7387570

[ref63] Schaufeli W. B. Salanova M. González-Romá V. Bakker A. B. (2002). The measurement of engagement and burnout: a two sample confirmatory factor analytic approach. J. Happiness Stud. 3, 71–92. doi: 10.1023/A:1015630930326

[ref64] Scott C. R. Connaughton S. L. Diaz-Saenz H. R. Maguire K. Ramirez R. Richardson B. . (1999). The impacts of communication and multiple identifications on intent to leave: a multimethodological exploration. Manag. Commun. Q. 12, 400–435. doi: 10.1177/0893318999123002

[ref65] Shan-huai L. (2022). Factors influencing the turnover intention of rural preschool teachers in the context of rural revitalization: an analysis based on a moderated mediation model. Best Evid. Chinese Educ. 11, 1523–1527. doi: 10.15354/bece.22.ab005

[ref66] Sharma U. Rajput B. (2021). Work engagement and demographic factors: a study among university teachers. J. Commerce Account. Res. 10:25.

[ref67] Shehzadi M. Khan A. A. (2024). The influence of workplace incivility on innovative work behavior: mediating effect of employee engagement and moderating effect of emotional intelligence. J. Asian Develop. Stud. 13, 495–511. doi: 10.62345/jads.2024.13.2.41

[ref68] Tayama J. Yoshida Y. Iwanaga R. Tokunaga A. Tanaka G. Imamura A. . (2019). Factors associated with preschool workers’ willingness to continue working. Medicine 97:e13530. doi: 10.1097/MD.0000000000013530, 30544456 PMC6310558

[ref69] Tran H. Winsler A. (2011). Erratum to “predictors of child protective service contact between birth and age five: an examination of California's 2002 birth cohort” [children and youth services review 33 (2011) 1337–1344]. Child Youth Serv. Rev. 33, 2399–2252. doi: 10.1016/j.childyouth.2011.07.008

[ref70] Trépanier S. Fernet C. Austin S. (2014). A longitudinal investigation of workplace bullying, basic need satisfaction, and employee functioning. J. Occup. Health Psychol. 20, 105–116. doi: 10.1037/a0037726, 25151460

[ref71] Tricahyadinata I. Hendryadi Suryani Zainurossalamia ZA S. Riadi S. S. (2020). Workplace incivility, work engagement, and turnover intentions: multi-group analysis. Cogent Psychol. 7:1743627. doi: 10.1080/23311908.2020.1743627

[ref72] Ullah R. Zada M. Saeed I. Khan J. Shahbaz M. Vega-Muñoz A. . (2021). Have you heard that—“GOSSIP”? Gossip spreads rapidly and influences broadly. Int. J. Environ. Res. Public Health 18:13389. doi: 10.3390/ijerph182413389, 34948998 PMC8704814

[ref73] Wang D. Niu Z. Sun C. Yu P. Wang X. Xue Q. . (2022). The relationship between positive workplace gossip and job satisfaction: the mediating role of job insecurity and organizational identity. Front. Psychol. 13:989380. doi: 10.3389/fpsyg.2022.989380, 36518950 PMC9742466

[ref74] Wartenberg G. Aldrup K. Grund S. Klusmann U. (2023). Satisfied and high performing? A meta-analysis and systematic review of the correlates of teachers’ job satisfaction. Educ. Psychol. Rev. 35:114. doi: 10.1007/s10648-023-09831-4

[ref9003] World Medical Association. (2013). World Medical Association Declaration of Helsinki: Ethical principles for medical research involving human subjects. JAMA, 310, 2191–2194. doi: 10.1001/jama.2013.281053 24141714

[ref75] Xu L. Feng B. Mao S. (2026). Negative workplace gossip and organizational citizenship behavior in the Chinese kindergarten teaching workforce: exploring interpersonal trust as a mediator. Front. Psychol. 17:1794504. doi: 10.3389/fpsyg.2026.1794504, 41937844 PMC13046526

[ref76] Yang F. Han Y. Li M. Y. (2021). If you believe, it may come true: the relationship and mechanism between self-occupation stereotypes of private kindergarten teachers and their turnover intention in China-mainland. Front. Psychol. 12:756099. doi: 10.3389/fpsyg.2021.756099, 34975647 PMC8718805

[ref77] Yang F. Yang H. (2023). The relationship between occupation stereotypes and turnover intention of private kindergarten teachers in China-mainland: a moderated mediation model. Am. J. Appl. Psychol. 12. doi: 10.11648/j.ajap.20231201.12 PMC8718805 34975647

[ref78] Zeng H. Zhao L. Li J. (2022). Why does subordinates’ negative workplace gossip lead to supervisor undermining? A moderated mediation model. Front. Psychol. 13:981539. doi: 10.3389/fpsyg.2022.981539, 36248567 PMC9559591

[ref79] Zhang Z. Li Y. Wang Y. An X. (2025). The influence of 8,786 Western China kindergarten teachers' emotional intelligence on work engagement. Front. Psychol. 16:1542911. doi: 10.3389/fpsyg.2025.1542911, 40201749 PMC11977667

[ref80] Zhang W. Meng H. Yang S. Liu D. (2018). The influence of professional identity, job satisfaction, and work engagement on turnover intention among township health inspectors in China. Int. J. Environ. Res. Public Health 15:988. doi: 10.3390/ijerph15050988, 29757985 PMC5982027

[ref81] Zhou X. Feng B. (2026). Psychological safety and Chinese PreK-12 teachers organizational citizenship behavior: the mediating role of interpersonal trust. Front. Psychol. 17:1786530. doi: 10.3389/fpsyg.2026.1786530, 41937812 PMC13043354

[ref82] Zou X. Chen X. Fengling C. Luo C. Liu H. (2020). The influence of negative workplace gossip on knowledge sharing: insight from the cognitive dissonance perspective. Sustainability 12:3282. doi: 10.3390/su12083282

